# Plasma cytokine and chemokine levels during natural outbreaks of bovine respiratory disease in young bulls on feedlots

**DOI:** 10.3389/fvets.2025.1617061

**Published:** 2025-07-09

**Authors:** Maud Rouault, Sébastien Assié, Blandine Gausserès, François Meurens, Gilles Foucras

**Affiliations:** ^1^Oniris, INRAE, BIOEPAR, Nantes, France; ^2^IHAP, Université de Toulouse, INRAE, ENVT, Toulouse, France; ^3^Research Group on Infectious Diseases in Production Animals (GREMIP) & Swine and Poultry Infectious Diseases Research Center (CRIPA), Faculty of Veterinary Medicine, University of Montreal, Saint-Hyacinthe, QC, Canada

**Keywords:** cytokine, cattle, BRD, bead-based assay, immunology, prognosis

## Abstract

Bovine Respiratory Disease (BRD) is a leading cause of morbidity and mortality in young cattle upon feedlot arrival. The immune response plays a dual role in infection control and lung tissue damage, but few studies have assessed cytokine levels during natural BRD outbreaks. Advances in multiplexed assays now allow for broader cytokine and chemokine profiling in cattle. In this nested case–control study, 184 young bulls from nine French fattening farms were clinically assessed and underwent thoracic ultrasonography (TUS) weekly during the first month on feed. BRD cases (*n* = 98) and matched healthy controls (*n* = 86) were selected based on clinical signs. Fifteen cytokines and chemokines were quantified in plasma using a bovine-specific bead-based multiplex assay, on the day an animal was first detected as sick and in its matched control sampled on the same day. BRD-associated pathogens were assessed using qPCR on nasal swabs and paired serology. The link between cytokines and clinical, microbiological, and preconditioning (vaccination and preventive antibiotic treatment) variables was investigated using the Wilcoxon-Mann–Whitney test, mixed-effects linear regression models, and multivariate clustering. Cytokine and chemokine levels did not distinguish clinically sick from healthy animals. No specific cytokine profile was associated with infection by a given pathogen. However, IL-17A and IFN-*γ* concentrations were positively associated with treatment relapse and disease severity, suggesting that they may have prognostic potential. Cluster analysis revealed three subgroups with distinct cytokine patterns and health outcomes, in association with preconditioning variables, highlighting the critical role of these interventions in shaping the immune response during BRD outbreaks. This study is the first to report the measurement of such a wide range of cytokines during spontaneous BRD episodes in young bulls. While not diagnostic when considered individually, cytokine profiles may hold prognostic value and could be integrated into multimodal risk stratification tools, in combination with clinical, microbiological data, and TUS results, to improve BRD management in the field. Preconditioning practices, such as vaccination or preventive antibiotic administration, significantly influence early immune responses and should further be investigated to refine prevention strategies and individualize health monitoring protocols at feedlot entry.

## 1 Introduction

Bovine Respiratory Disease (BRD) represents a major health issue in both dairy and beef cattle, leading to significant economic losses (1). Its occurrence results from a complex interaction between viral and bacterial pathogens, host-related factors—including immunity—and environmental conditions, notably stress-related factors such as weaning, commingling, and transportation (2, 3). Pathogens act synergistically, with the most commonly described scenario of BRD development involving a primary viral infection followed by a bacterial superinfection, although some bacteria may also serve as primary causative pathogens of BRD (3, 4). The viral pathogens most frequently associated with BRD include *Orthopneumovirus bovis* (*Bovine Respiratory Syncytial Virus,* BRSV), *Respirovirus bovis* (*Bovine Parainfluenza 3 Virus,* BPI3V), Var*icellovirus bovinealpha1* (*Bovine Herpesvirus 1,* BoHV-1), *Pestivirus bovis* (*Bovine Viral Diarrhea Virus,* BVDV), *Betacoronavirus gravedinis* (*Bovine Coronavirus*, BCoV), and *Deltainfluenzavirus influenzae* (*Influenza D Virus,* IDV) (3). Notably, the latter two viruses have been more recently linked to BRD, and their exact role in disease pathogenesis remains unclear (4). The bacterial species most commonly isolated from cattle with BRD are often commensal organisms of the upper respiratory tract, namely *Mannheimia haemolytica, Pasteurella multocida* and *Histophilus somni* (3). In addition, *Mycoplasmopsis bovis* is an important opportunistic pathogen whose prevalence in feedlot cattle increases significantly after transportation (5).

The immune system plays a key role in the development of BRD, as it is central for clearing infections caused by pathogens, but can also contribute to lung tissue damage, particularly through the side effects of the neutrophil response (2). Given its dual role, the immune response to experimental infections or co-infections with BRD-associated pathogens has been the focus of numerous studies, particularly through the measurement of cytokine production or gene expression in the plasma or cells collected after bronchoalveolar lavage (6–15). These studies have provided valuable insights into the immune mechanisms associated with a BRD episode. However, most were conducted under controlled experimental conditions with inoculation of a known pathogen and therefore failed to capture the full complexity of interactions between pathogens, environmental factors, and animal history. In contrast, very few studies have examined cytokine production during a natural BRD outbreak (16–19). Among them, two studies reported increased concentrations of IL-1β, IFN-*γ*, IL-8 and TNF-*α* in the first study, and IL-17A and TNF-α in the second, in animals affected by BRD of confirmed bacterial origin compared to controls (16, 19). In contrast, two other studies reported variations in cytokines (IFN-*γ*, IL-1β, IL-6 and TNF-*α* in the first study, and IL-8, IL-1β, and TNF-α in the second) depending on factors such as duration on feed, farm of origin, and number of treatments. However, cytokine concentrations alone did not discriminate cases from controls, which were defined based on a clinical examination alone (17, 18). These findings highlight the need for further investigation to determine whether cytokine profiling during natural BRD outbreaks could have diagnostic or prognostic value. In addition, cytokine measurement in cattle has improved with the recent development of multiplexed assays that allow the determination of innate and adaptive cytokines and major chemokines, which was not technically possible until recently.

In this context, the present study had two main objectives. First, we sought to compare the concentrations of 15 cytokines and chemokines measured using a 15-plex bead-based assay recently developed and validated in cattle (20), between healthy and diseased young cattle on arrival at feedlots. Secondly, we aimed to identify the relationship between health and history factors that may influence cytokine concentrations, thereby contributing to a more comprehensive understanding of the immune response in naturally occurring BRD.

## 2 Materials and methods

### 2.1 Enrollment of cases and controls

Between January and December 2023, 224 young bulls from nine fattening farms in Western France were monitored for BRD for one month after their arrival. The bulls were retrieved from various cow-calf farms across France at the beginning of the week and brought to a sorting facility, where they were grouped based on weight and age. By the end of the week, they were delivered to the fattening farms (D0). They were then raised in batches, corresponding to a group of eight to twelve bulls housed together in the same pen on the farm. A total of 20 batches (one to three per farm) were monitored during the period.

Upon arrival or at the sorting facility, the bulls could receive deworming treatment (with macrocyclic lactones) and vaccination against respiratory pathogens. Thus, the vaccination program was consistent within each farm but varied between farms. Two types of vaccines were used: an intranasal vaccine containing modified-live viruses (MLV) for BRSV and BPI3V (Rispoval RS + PI3 Intranasal, Zoetis, Malakoff, France) and a subcutaneous vaccine containing killed viruses (KV) for BRSV, BPI3V and *Mannheimia haemolytica* (either Bovalto Respi 4, Boehringer Ingelheim, Lyon, France, or Bovilis Bovigrip, Intervet, Beaucouzé, France). Seven out of nine farms used only the KV vaccine. One farm used the MLV intranasal vaccine, followed by the KV vaccine 10 days later. One farm did not vaccinate.

Young bulls were clinically assessed on D5, D14, D21, and D28 (+/− 1 or 2 days, depending on the farmer’s availability) by the same veterinarian. Clinical examinations included rectal temperature measurement and visual signs of BRD (e.g., nasal or ocular discharge, coughing, increased respiratory rate, dyspnea, decreased rumen fill, or depression). All young bulls also underwent thoracic ultrasonography (TUS) of the fourth and fifth intercostal spaces (ICS), either on both sides (six farms) or only on the left (two farms) or right side (one farm) of the thorax, depending on restraint conditions at the farm. The largest consolidation (≥ 1cm^2^) (21) observed in these ICS for each animal was recorded on every examination day.

**Figure 1:**
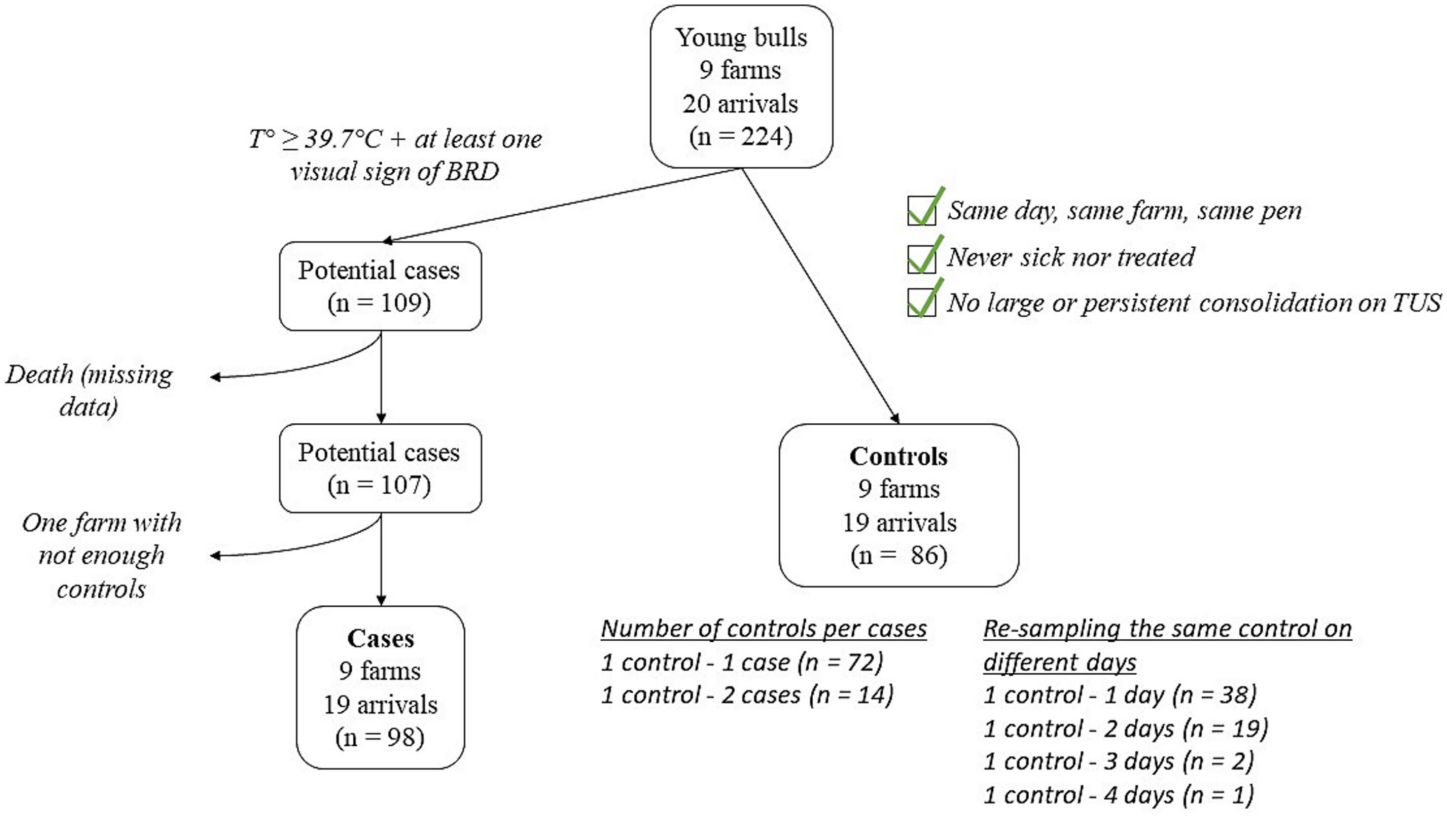
Flowchart depicting the selection of BRD-clinically affected cases and matched controls.

An animal was considered as a potential case on the first day when its rectal temperature was equal to or greater than 39.7°C, and it exhibited at least one clinical BRD sign. Controls were selected at the end of the follow-up month, as described in Figure 1. Briefly, controls originated from the same farm and the same pen as their matching cases, and they were sampled on the same day. Additionally, a control animal could not have been detected as sick, nor have been treated at any point during the study, and could not have exhibited large (> 1cm^2^) or persistent (> 1 examination day) consolidation on TUS. The number of controls per case and the number of controls re-sampled on different examination days are given in Figure 1. Eighty-six controls were included in the study. Among the potential cases, two were excluded because of missing data (animals died before the end of the study), and nine animals from the same farm were excluded because no matching controls could be included. In total, 98 cases were eventually included.

Antibiotic treatments were decided by the farmer, including both the decision to treat and the choice of molecule, and were recorded (treatment = 0, 1, ≥2). Similarly, the number of days the animal was observed as sick was recorded (sickness count = 0, 1, ≥2).

### 2.2 Pathogen screening

Two nasal swabs (one in each nostril), using sterile 175-mm-long swabs, were taken from cases. After collection, they were immediately placed on ice and transported to the laboratory within 3 h, where both swabs from the same animal were suspended in the same 400–600 μL of phosphate-buffered saline (PBS), and frozen at −80°C (−112°F) until analysis. qPCR analyses were conducted within a maximum of 6 months after freezing at the BIOEPAR laboratory (Nantes, France) using various commercial kits (BIOTK051, BIOTK052, BIOTK053, BIOTK054, BioSellal, Dardilly, France) to detect *Mannheimia haemolytica* (*M. haemolytica*), *Pasteurella multocida, Histophilus somni, Mycoplasmopsis bovis* (*M. bovis*), *Orthopneumovirus bovis* [Bovine Respiratory Syncytial Virus (BRSV)], *Respirovirus bovis* [Bovine Parainfluenza Virus type 3 (BPI3V)], *Betacoronavirus gravedinis* [Bovine Coronavirus (BCoV)], and *Deltainfluenzavirus influenzae* [Influenza D Virus (IDV)].

Additionally, blood samples were collected from all animals on D0 and D28 via coccygeal venipuncture using plain tubes. After collection, the blood was left to clot at room temperature before being centrifuged at 1000 g for 10 min at 4°C (39°F). The resulting serum was then frozen at −20°C (−4°F) until analysis. Serological analyses were conducted within 6 months by Intervet International B. V (Boxmeer, Netherlands) using two commercial ELISA kits (BIO K 392/2, BIO K 369/2, BioX Diagnostics, Rochefort, Belgium). Seroconversion was assessed for *M. haemolytica, M. bovis*, BRSV, BPI3V, and BCoV. According to the manufacturer’s recommendations, seroconversion was defined as at least a two-fold increase in the test result for the same animal between D0 and D28. Antibody levels of four or five crosses at D0—five being the maximum value reported by the kits—were interpreted as ‘high antibody levels at D0’.

The infection status of the animal regarding each BRD pathogen was determined by evaluating qPCR results, antibody levels at D0 and D28, and the vaccination protocol used on each farm, as the latter could influence the seroconversion or qPCR results (22). For each pathogen (*M. haemolytica, M. bovis,* BRSV, BPI3V and BCoV), animals were considered ‘immune’ if they had a positive qPCR result and had received intranasal vaccination against that specific pathogen, or if they had seroconverted after receiving subcutaneous vaccination, or if their antibody level at D0 was high. Animals were considered ‘infected’ if they had a low antibody level at D0 and a positive qPCR result or seroconversion for a pathogen they had not been vaccinated against. All other animals were classified as ‘neither immune nor infected’.

### 2.3 Cytokines and chemokines assessment

Blood samples (4 mL; Heparin Lithium, BD Vacutainer) were collected by coccygeal venipuncture on all animals on all examination days. After collection, they were immediately placed on ice and transported to the laboratory within 3 h, where they were centrifuged at 1000 g for 10 min at 4°C (39°F). The resulting plasma was frozen at −80°C until analysis, which was conducted within 10 months. Plasma concentrations for 15 cytokines (IL-1*α*, IL-1β, IL-1RA, IL-2, IL-4, IL-6, IL-17A, IFN-*γ*, CCL2, CCL3, CCL4, CXCL8, CXCL10 and TNF-α) were determined using a custom bovine cytokine/chemokine bead-based multiplex assay (SPRCUS617, Milliplex® xMAP®, Merck-Millipore, France) (20). Briefly, plasma samples were carefully resuspended after thawing and centrifuged for 5 min at 3000 g to remove debris. After a 1:2 dilution, they were assayed according to the supplier’s instructions. Mean Fluorescence Intensity (MFI) were recorded on a MaGPix system and processed using the xPONENT Software (Luminex) to determine cytokine concentrations using internal analyte references. Cytokine concentrations under the lower limit of detection (LLOD) were all set to the LLOD value.

### 2.4 Statistical analysis

All statistical analyses were conducted using RStudio (RStudio: Integrated Development Environment for R. Posit Software, PBC, Boston, MA, version 4.3.1). Results were considered significant if *p* < 0.05.

To assess the normality of data, the Shapiro–Wilk test was applied. Differences in cytokine and chemokine levels between the two groups of animals (cases *vs.* controls) were then assessed using the Wilcoxon-Mann–Whitney test.

Due to the skewed distribution of cytokines in the study population, the data were unsuitable for analysis using linear regression methods. To address this, cytokine concentrations were log10-transformed to approximate a normal distribution. Separate linear mixed-effects models were then built for each log10-transformed cytokine as the dependent variable. The independent linear variables (rectal temperature, arrival weight and arrival age) were transformed into categorical variables since none of them were linearly associated with cytokine concentrations. The first quartile, median, and third quartile values were used to define four categories for each of these variables. Independent variables included health related data such as sickness count (0, 1, ≥2), treatment (0, 1, ≥2), rectal temperature (<39.3°C, [39.3–39.7°C], [39.7–40°C], ≥40°C), TUS results (consolidation ≥ 1cm^2^ = no, yes) and infection status for the five pathogens (‘neither immune nor infected’, ‘immune’, ‘infected’). As only one of the cases was considered ‘immune’ for *Mycoplasmopsis bovis*, this variable was ultimately treated as binary (‘not infected’, ‘infected’). They also included conditioning data, namely treatment on D0 (no, yes), vaccination on arrival (no, yes), arrival weight (<293 kg, [293-322 kg], [322-355 kg], ≥355 kg), arrival age (<8 months, [8-9 months], [9-10 months], ≥10 months). Rectal temperature was ultimately not included in the analyses as it was collinear with sickness count, given that temperature was used to define diseased animals. Categorical variables were coded with the first modality as the reference. The batch was included as a random effect. To refine the models, a stepwise backwards selection procedure was applied after fitting the models. Variables were progressively removed based on their contribution, guided by the Akaike Information Criterion. The final models retained only variables that significantly explained cytokine concentrations. The quality of the linear mixed-effect models was assessed by verifying the normality and homoscedasticity of residuals, as well as the absence of multicollinearity among independent variables.

Cytokines for which the quality of the linear mixed regression model was satisfactory and that showed a significant association with at least one independent variable or with the random effect (batch) were selected for a Factorial Analysis of Mixed Data (FAMD) to identify different cytokine profiles in relation to health and conditioning data. The FAMD was performed using the Factoshiny interface from the FactoMineR package. A Hierarchical Clustering on Principal Components was then conducted subsequently. For this analysis, linear variables (rectal temperature, arrival age, arrival weight) were used without modifications.

## 3 Results

### 3.1 Plasma cytokine and chemokine concentrations are not different between clinically sick BRD cases and healthy controls on feed

The main characteristics of the recruited animals are presented in Table 1. A total of 184 young bulls were included in the study (98 cases and 86 controls). They were predominantly of Charolais (149/184) and Limousine (20/184) breeds, with a mean age of 9.0 months [standard deviation (SD) = 1.8, range: 6–14 months], and a mean weight of 322.8 kg (SD = 44.0, range: 224–482 kg). No significant differences in breed, age and weight were found between the two groups.

**Table 1:** Characteristics of cases (BRD-clinically affected) and controls (healthy) in 184 young bulls followed-up during the first month of fattening in nine fattening farms.

Factors	Controls (*n* = 86)	Cases (*n* = 98)
Preconditioning-related factors		
Age (months)		
< 8	15	23
[8–9]	26	24
[9–10]	15	17
≥ 10	30	34
Weight (kg)		
< 293	17	29
[293–322]	20	23
[322–355]	27	21
≥ 355	22	25
Breed		
Charolais	83.7% (72)	78.6% (77)
Limousine	9.3% (8)	12.2% (12)
Others	7.0% (6)	9.2% (9)
Vaccination		
Vaccinated animals	77.9% (67)	77.5% (76)
Treatment on D0		
Treated animals	22.1% (19)	22.4% (22)
Health-related factors		
Rectal temperature (°C)	39.1°C (SD: 0.4, range: 38.0–39.8)	40.1°C (SD: 0.5, range: 38.9–41.8)
Sickness count		
Never sick	100% (86)	0%
Sick once	0%	65.3% (64)
Sick twice or more	0%	34.7% (34)
Treatment		
Never treated	100% (86)	55.1% (54)
Treated once	0%	30.6% (30)
Treated twice or more	0%	14.3% (14)
TUS results		
Animals with at least one consolidation	1.2% (1)	27.5% (27)
Pathogens		
*Mannheimia haemolytica*		
Neither infected nor immune	22.1% (19)	35.7% (35)
Immune	70.9% (61)	38.8% (38)
Infected	7.0% (6)	25.5% (25)
*Mycoplasmopsis bovis*		
Not infected	57.0% (49)	42.9% (42)
Infected	43.0% (37)	57.1% (56)
BRSV		
Neither infected nor immune	43.0% (37)	34.7% (34)
Immune	55.8% (48)	56.1% (55)
Infected	1.2% (1)	9.2% (9)
BPI3V		
Neither infected nor immune	43.0% (37)	35.7% (35)
Immune	53.5% (46)	53.1% (52)
Infected	3.5% (3)	11.2% (11)
BCoV		
Neither infected nor immune	40.7% (35)	24.5% (24)
Immune	5.8% (5)	2.0% (2)
Infected	53.5% (46)	73.5% (72)

Among the cases, 65.3% (64/98) were detected as clinically sick once during the first month on feed, while 34.7% (34/98) were detected clinically ill twice or more. One control animal had a 1cm^2^ consolidation, while 27.5% (27/98) of the cases developed at least one consolidation (≥1cm^2^) during the follow-up period. Antibiotic treatment was administered once to 30.6% (30/98) of the cases and at least twice to 14.3% (14/98) of the cases. Preventive treatment administration with tildipirosin on D0 was practiced in two farms. Sick animals were mostly administered with florfenicol (six farms) or tulathromycin (one farm) at first detection. In two farms (with only one batch of animals included in the study per farm), no antibiotic was administered at all. Second-line treatments were most frequently carried out with a combination of lincomycin and spectinomycin, tulathromycin, tildipirosin, or oxytetracycline. In most cases, at least during the first antibiotic treatment, the bulls also received a non-steroidal anti-inflammatory drug (flunixin, meloxicam, tolfenamic acid). The anti-inflammatory treatment was sometimes repeated during subsequent antibiotic treatments.

**Figure 2:**
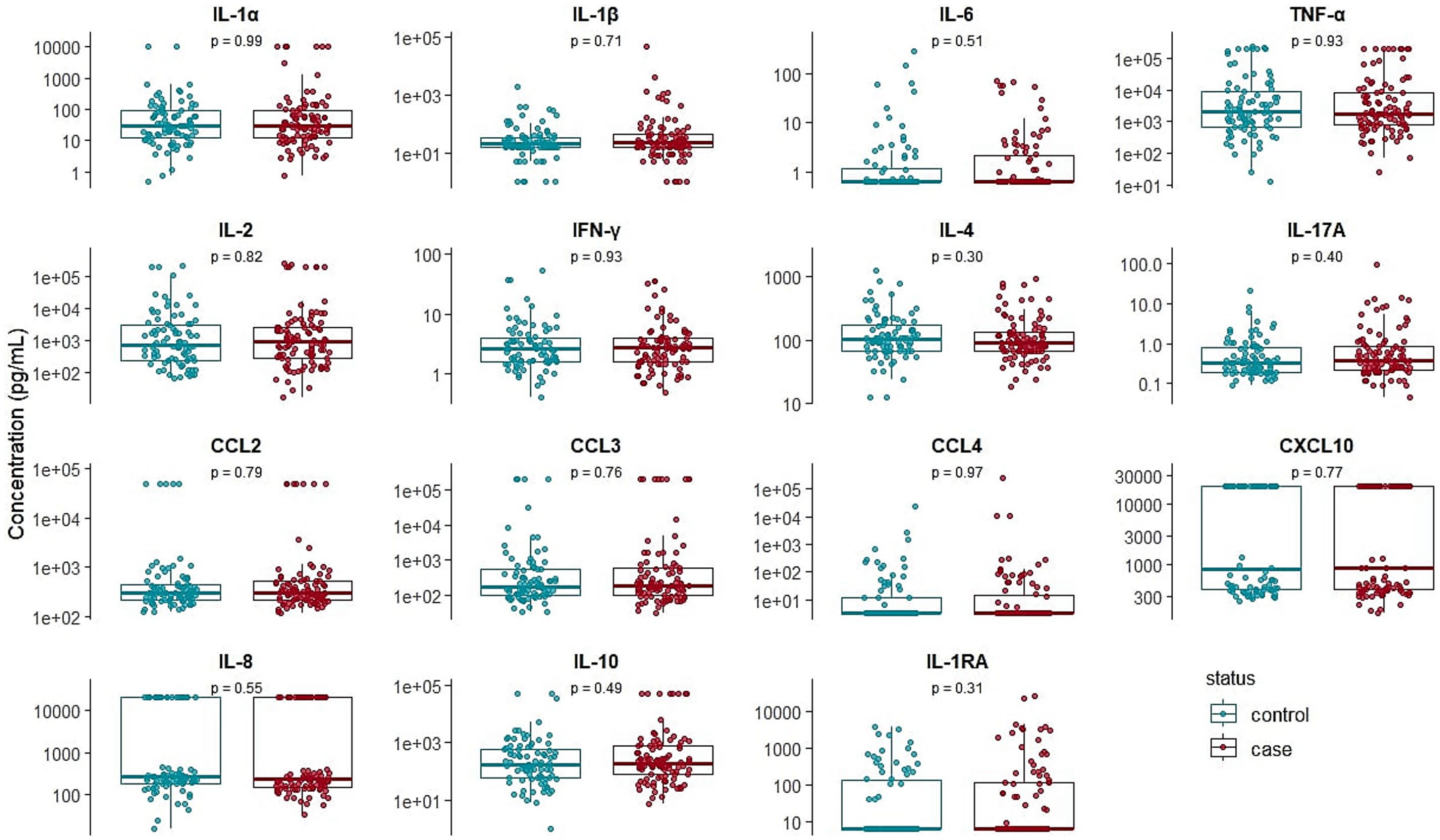
Box plots comparing the variability of IL-1*α*, IL-1β, IL-1RA, IL-6, IL-17A, IL-2, IL-4, IFN-*γ*, IL-8, CXCL10, CCL2, CCL3, IL-10, CCL4, and TNF-α between clinically healthy (*n* = 86) and bovine respiratory disease-affected (*n* = 98) young bulls during the first month on feed. *p*-values are coming from the Wilcoxon-Mann–Whitney test.

The plasma levels of IL-1*α*, IL-1β, IL-1RA, IL-6, IL-17A, IL-2, IL-4, IFN-*γ*, CXCL8, CXCL10, CCL2, CCL3, IL-10, CCL4, and TNF-α are presented in Figure 2. No significant differences were observed in the levels of any cytokines or chemokines between cases and controls based on the clinical definition of BRD, indicating that cytokine measurement is insufficient to distinguish BRD-affected animals.

### 3.2 Cytokine expression is not linked to BRD etiology but a few cytokines measured at the time of first detection may serve as prognostic indicators

We next investigated if individual cytokines correlated with pathogen detection, health status, or management-related parameters.

No significant association was found between any of the cytokines and the infection status for the five pathogens that were assessed through qPCR or serology (*M. bovis, M. haemolytica,* BRSV, BCoV, and BPI3V), arrival weight, age, or vaccination status. Likewise, no significant association was found for IL-1*α*, IL-2, IL-10, CCL2, CCL3, CXCL8, CXCL10 and TNF-α with any of the independent variables included in these models.

Table 2 presents the significant results of the linear mixed-effects models after stepwise backwards selection, along with the variance in each cytokine concentration explained by the selected variables. Several cytokines were significantly associated with health parameters (sickness count, treatment, TUS consolidation) and/or pre-conditioning data (treatment on D0, arrival weight, arrival age). Only the results of models deemed to be of sufficient quality were presented (assessing the normality and homoscedasticity of residuals). Thus, IL-1RA and IL-6, although associated with the random effect after stepwise backwards selection, were not retained for further analysis. Similarly, CCL4, although negatively associated with treatment on D0, was not kept in the downstream analysis.

**Table 2:** Effect of health and conditioning data on four cytokine concentrations using linear mixed-effects models with stepwise backward selection in 184 young bulls during the first month of fattening.

Predictor variables	IL-1β	IL-17A	IL-4	IFN-γ
Health				
Sickness count				
≥2		0.34 (0.14)*		
Treatment				
≥2				0.70 (0.20)***
TUS Results				
Consolidation_yes		−0.29 (0.14)*		−0.38 (0.15)*
Pre-conditioning				
Treatment on D0				
Treatment D0_yes		−0.31 (0.11)**		
Arrival weight				
[322–355 kg]			−0.44 (0.17)**	
≥355 kg			−0.50 (0.19)**	
Arrival age				
[9–10 months]			0.45 (0.18)*	
≥ 10 months			0.38 (0.16)*	
Random effect (batch)	0.33 (0.57)		0.09 (0.30)	
Final model R^2^	15.9	6.8	23.7	7.1

The partial slope coefficients for the fixed effects in the final models are presented with their standard deviations (in parentheses). The variance and standard deviation (in parentheses) of the random effect are reported only if the random effect remains in the final model after stepwise backward selection. The R^2^ value (R^2^c when the random effect was kept in the final model or R^2^m when it was not) estimates the proportion of variance in cytokine concentration explained by the final model. COND, Conditioning. *p < 0.05, **p < 0.01, ***p < 0.001.

IL-17A concentration was positively associated with a sickness count ≥ 2 and was negatively associated with the presence of TUS consolidation and antibiotic treatment on D0. IL-4 concentration was positively associated with higher age categories but negatively associated with higher weight categories. Finally, IFN-*γ* concentration was positively associated with a number of treatments ≥ 2 and negatively associated with TUS consolidation.

For IL-1β, only the random effect (batch effect) remained in the final model, explaining a significant portion of the variance in cytokine concentrations (15.9%). Nevertheless, the overall variance in cytokine concentrations explained by the final models was low (2.1–26.3%), suggesting that cytokine concentrations are also influenced by several other factors not included in these models.

### 3.3 Cattle subgroups with different health and preconditioning statuses are identified through cytokine profiling

Since a few cytokines correlated with health and management indicators, we considered them together to see if we could identify subgroups of cattle with differences in BRD severity or outcome. IL-17A, IFN-*γ*, IL-1β, and IL-4 were included in a Factorial Analysis of Mixed Data (FAMD), along with health-related variables (sickness count, treatment, rectal temperature, and infection status regarding the five pathogens) and preconditioning variables (arrival weight and age, vaccination on D0, treatment on D0). The first 10 principal component axes, each with an eigenvalue greater than 0.99 and together explaining 72.7% of the total variance, were retained for the analysis. The two most contributing dimensions explained, respectively, 13.9 and 11.8% of the total variance.

**Figure 3:**
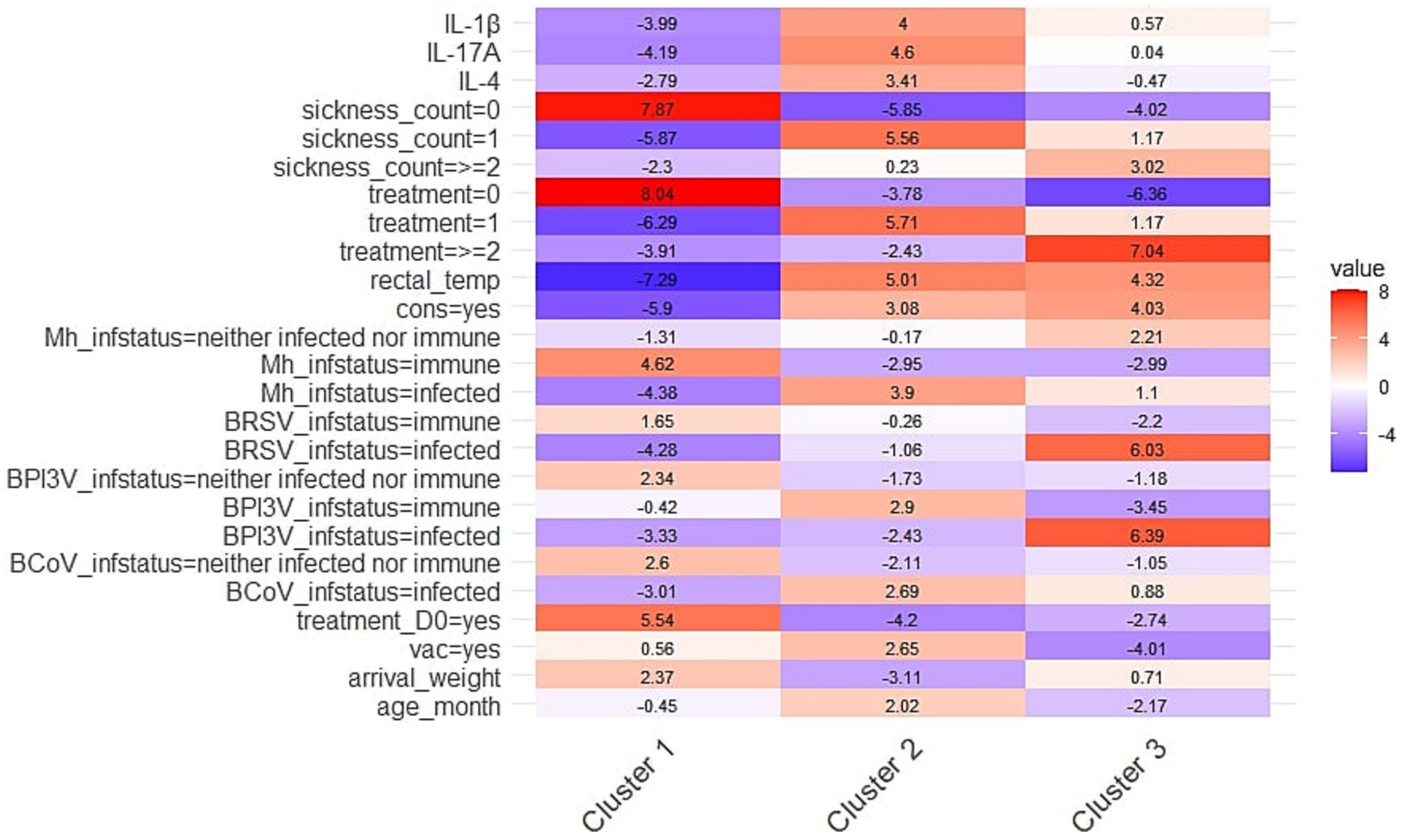
Heatmap representing the standardized variations (v.test) of the health-related (cytokine levels, sickness count, treatment, rectal temperature, TUS-consolidation, infection status) and conditioning variables (treatment on D0, vaccination, arrival weight, arrival age) significantly associated with cluster definition, relative to the overall population. Clusters were obtained by Hierarchical Clustering on Principal Components conducted after Factorial Analysis of Mixed Data in 184 young bulls exposed to natural outbreaks of BRD during the first month on feed. Positive values (in red) indicate that a variable is more prevalent in a given cluster than expected, while negative values (in blue) indicate lower prevalence. Rectal_temp, rectal temperature; cons, consolidation on TUS, Mh, *Mannheimia haemolytica*, BRSV, *Bovine Respiratory Syncytial Virus* (*Orthopneumovirus bovis*); BPI3V, *Bovine Parainfluenza 3 Virus* (*Respirovirus bovis*); BCoV, *Bovine Coronavirus* (*Betacoronavirus gravedinis*); vac, vaccination.

The Hierarchical Clustering on Principal Components identified three clusters. Figure 3 shows the standardized variations (v.test) of the variables significantly associated with cluster definition, relative to the overall population.

Cluster 1 included 119 young bulls, with a significantly higher proportion of healthy animals exhibiting a normal rectal temperature, no BRD treatment, and no TUS consolidation compared to the overall study population. This cluster also had fewer animals with an ‘infected’ status for *M. haemolytica*, BCoV, BPI3V and BRSV. Additionally, a greater proportion of animals in this group received treatment on D0, and the mean arrival weight was higher than in the rest of the study group. Finally, IL-4, IL-1β, and IL-17A levels were significantly lower in this group.

Conversely, Cluster 2, which included 46 bulls, was characterized by significantly higher levels of IL-4, IL-1β and IL-17A compared to the overall population. Animals in this group were predominantly diagnosed as sick once, received BRD treatment once and exhibited TUS consolidation. This cluster also had a significantly higher proportion of individuals with an ‘infected’ status for *M. haemolytica*, BPI3V, and BCoV. Additionally, mean rectal temperature was higher in this group. Regarding pre-conditioning variables, most animals had been vaccinated but had not received treatment on D0. They were also older on arrival but had a lower body weight compared to the overall population.

Finally, Cluster 3, which included 19 bulls, was characterized by a high proportion of animals diagnosed as clinically sick at least twice, having received at least two BRD treatments, exhibiting TUS consolidation and having an elevated rectal temperature (mean = 40.3°C). This cluster also had significantly more individuals classified as ‘infected’ with BRSV and BPI3V compared to the overall population. Furthermore, the majority of this group had not received any vaccination or treatment on D0. They were also the youngest of the group. Notably, cytokine levels did not significantly contribute to the definition of this cluster.

## 4 Discussion

In this study, we assessed the plasma concentrations of 15 cytokines and chemokines using a multiplex bead-based assay in clinically BRD-affected and healthy matched controls during their first month on feedlots. To the best of our knowledge, this is the first study to evaluate such a broad range of cytokines and chemokines in natural BRD cases. Individual cytokine levels failed to discriminate between clinically sick and healthy animals. These findings are consistent with two previous studies that evaluated a smaller panel of cytokines (IL-1β, IL-8 and TNF-*α*, and IL-1β, TNF-α, IL-6, and IFN-*γ*) during natural BRD outbreaks in stocker calves and feedlot cattle (17, 18). As in our study, animals were not monitored daily for BRD clinical signs. Instead, sampling occurred at specific time points—once a week (D0, D7, D14, D21) in the first study (17) and on four occasions (D0, D1 [feedlot arrival], D9, D28) in the second (18). This limited number of samplings may have failed to capture the transient and time-dependent expression of cytokines throughout the course of BRD. Indeed, experimental infections with BRD-associated viral and bacterial pathogens have shown that cytokine responses are highly dynamic and variable over time with broad and heterogeneous peaks among infected animals (10, 23, 24). Another explanation for the absence of significant differences in cytokine and chemokine levels between healthy and sick animals may lie in the method used to identify BRD cases. Animals were classified as cases based solely on clinical BRD signs (e.g., nasal or ocular discharge, coughing, increased respiratory rate, dyspnea, decreased rumen fill, or depression) and rectal temperature. However, a systematic review and meta-analysis reported that clinical signs observed by pen checkers in feedlot cattle have a low to very low sensitivity of detection (0.27) when compared to lung lesions recorded at slaughter (25). Even when using clinical scoring in pre-weaned dairy calves, the sensitivity of the scores remains relatively low (0.46) when compared to TUS abnormalities (26). Although TUS was performed in this study, it was not used to define cases, as its diagnostic performance, especially in the early stages of BRD, remains unknown in young bulls. Consequently, it was not included in the case definition. As a result, case recruitment may have included animals with upper respiratory tract infections rather than true broncho-pneumonia. Indeed, only 27.5% of clinical cases in this study exhibited consolidation on TUS, which is consistent with findings from another study on the same category of animals (27). In animals older than 6 months, like the young bulls of our study, the cranial parts of the cranial pulmonary lobes are not accessible to TUS due to the forelimb musculature. This likely reduces the sensitivity of detection with TUS in these animals compared to pre-weaned calves. BRD in cattle typically begin in the cranial part of the right cranial lobe (21, 28). Nevertheless, as the sensitivity and specificity of TUS for detecting lung lesions in young bulls are unknown, we cannot rule out the inclusion of animals with upper respiratory tract infections among our cases, without comprehensive data on how cytokines vary in such infections. However, in the study by Akter et al. (17), no association was found between health status, defined by TUS results, and cytokine levels.

Moreover, we did not include the nature of pathogens that were detected, particularly bacteria such as *M. haemolytica* and *M. bovis*, in the definition of cases (or the exclusion of animals carrying these bacteria in the definition of controls), as was done in two other studies that observed significant differences in cytokine concentrations during natural BRD outbreaks (16, 19). In the first study, confirmed cases were clinically affected by BRD, and *M. haemolytica* or *H. somni* were identified by culture on nasopharyngeal swabs. Controls were clinically healthy animals, with no pathogens detected by qPCR (BRSV, BPI3V, BVDV, BoHV-1) or culture (*M. haemolytica* and *H. somni*) (19). In the second study, cases were defined by severe clinical signs (dyspnea, tachypnea), abnormal complete blood count, and the isolation of *M. haemolytica*, *P. multocida*, or *M. bovis* in culture on the bronchoalveolar lavage fluid (BALF). Controls were clinically healthy, without CBC changes, and they also tested negative for pathogens in BALF (16). It is therefore likely that, in natural BRD outbreaks, cytokine variations may be detectable only transiently, or more persistently in cases of severe pneumonia, particularly bacterial pneumonia, which is known to cause extensive and sometimes severe inflammatory lung lesions (28–30). In contrast, no pathogen was detected in 11 clinically BRD-affected animals, and they were considered not infected according to our definition of infection status, whereas 65 cases were infected with at least one pathogen. While this could be attributed to the way infection status was defined—to account for differences in vaccination protocols across farms—it likely underestimated true infection cases by any of the searched pathogens against which animals were not vaccinated, specifically *M. bovis* and BCoV. Notably, these two pathogens were the most prevalent among control animals (43.0 and 53.5%, respectively), although they were still less prevalent than in cases. It is worth noting that *M. bovis* can act as an opportunistic pathogen present in the nasal cavities of clinically healthy animals (31), and the exact role of BCoV in BRD pathogenesis remains unclear (4). However, adding pathogen information to the definition of cases and controls may not have been appropriate, as it could have unbalanced the groups and compromised the ability to match animals by farm. Since the farm of origin can influence the cytokine background, as shown by Chitko-McKnown et al. for IL-6, with a similar trend observed for TNF-*α*, matching case and control at the farm level was a key priority in our study design. A comparable association was also observed in our dataset, as highlighted by linear regression analyses showing a significant effect of farm on several cytokines or receptors, namely IL-1β, IL-4, IL-1RA, and IL-6. However, IL-1RA and IL-6 were excluded from further analyses due to poor model performance, driven by violations of residual assumptions (non-normality and/or heteroscedasticity). These issues likely stemmed from the non-normal distribution of these cytokines, with concentrations frequently falling below the lower detection limit.

To the best of our knowledge, this is also the first study to investigate potential associations between cytokine levels and infection by specific pathogens, both bacterial and viral, during natural outbreaks of BRD. In human medicine, certain cytokines such as IL-8, TNF-*α*, and IL-10 have recently been used to differentiate *Mycoplasma pneumoniae* infection from viral pneumonia in children (32). However, in the present study, no significant association was found between cytokine levels and the presence of a specific pathogen in the linear regression models. This is consistent with the complexity of naturally occurring infections, where co-infections involving at least two pathogens accounted for approximately 60% of all infections in the study, thereby making it difficult to identify cytokine profiles that are specifically associated with individual bacterial or viral pathogens. Indeed, increased expression or concentrations of pro-inflammatory cytokines involved in the innate immune response such as IL-1*α*, IL-1β, IL-6, and TNF-α, have been reported in experimental *in vivo* or *in vitro* infections caused by both bacteria like *M. haemolytica* (7, 11, 33) and *M. bovis* (34), or viruses as BoHV-1 (7), BRSV (10, 15, 35), and BVDV (23). Similarly, expression of IL-4, a cytokine associated with type 2 responses, has been shown to increase during experimental infection with BRSV (9, 12, 15), as well as *M. bovis* (34). CXCL-8 (IL-8), is a key chemokine involved in neutrophil recruitment (2), that triggers tissue injury during pneumonic pasteurellosis, and is overexpressed not only in *M. haemolytica* infections (36–38), but also in response to BRSV infection (14). IL-17, a pro-inflammatory cytokine linked to type 3 immunity, that stimulates IL-8 production, has been shown to rise following *in vivo* infection with BRSV or *M. haemolytica* (38). Taken together, these findings suggest that no specific cytokine profile reliably distinguishes infections caused by individual BRD pathogens, or discriminate between bacterial and viral infections, with the possible exception for type I interferons (IFN-*α* and IFN-*β*), which are more specifically involved in the host response to viruses (39), although they have also been associated with certain bacterial infections (40). These interferons were not directly measured in our study, but CXCL10 was used as a proxy marker of interferon activity, although no association with viral infections was detected. While cytokine and chemokine levels do not allow for differentiation between specific etiologies in the context of natural BRD outbreaks, they could still be integrated into composite scoring systems that include additional clinical or imaging data, where they may provide added value, similarly to what has been developed in human medicine (32, 41).

Rather than aiding in diagnosing BRD or indicating a specific etiology, two cytokines in this study seem to have prognostic value. Indeed, as shown by linear regression (Table 2), elevated IL-17A was positively associated with a relapse after the first treatment. Similarly, IFN-*γ* concentration was positively associated with the recourse for more than two treatments. These findings are particularly noteworthy since elevated levels of these cytokines were observed at the first instance of illness. Akter et al. reported the same link regarding TNF-*α* levels, which were higher in animals requiring two treatments. A transcriptomic study also reported an upregulation of genes associated with complement factor B, pro-inflammatory responses (*LRG1, MCF2L, SPP1*), and type I interferons (*HERC6, IFI6, ISG15, MX1*) in animals that ultimately required more than two antimicrobial treatments (42).

Finally, the clustering analysis identified an interesting association between cytokine profiles (IL-17A, IL-1β, and IL-4) and health-related variables linked to health status or preconditioning. One cluster, composed predominantly of healthy animals that tested negative for all investigated pathogens, was characterized by significantly lower concentrations of IL-17A, IL-1β and IL-4 compared to the whole group. This subgroup included a higher proportion of animals that had received metaphylactic antibiotic treatment on D0. This finding is not unexpected, as this treatment was administered with tildipirosin, a macrolide antibiotic known for its immunomodulatory properties, particularly its ability to reduce the levels of several cytokines (18, 43). The two other clusters were composed of clinically sick animals, characterized by elevated rectal temperatures, detection of respiratory pathogens, and TUS consolidation. In one of these clusters, a favorable response to infection was observed, as animals recovered rapidly and required only a single treatment. This cluster included vaccinated animals and was associated with elevated concentrations of IL-17A, IL-1β and IL-4 compared to the overall study population. This observation is in favor of vaccination against BRD pathogens and contrasts with the conclusions of several reviews that have reported inconsistent or limited benefits of vaccination at feedlot arrival (29, 44). The second cluster consisted of more severely affected animals, characterized by delayed or absent healing. These animals had neither received metaphylactic treatment nor vaccination on D0, and their cytokine concentrations were moderate, neither significantly higher nor lower than the group average. These findings underscore the importance of accounting for preconditioning status when interpreting cytokine profiles, an aspect not highlighted in previous studies, either due to insufficient sample size (18) or because the treatment and vaccination protocols were the same for all animals included in the study (19). This is particularly relevant for IL-17A, which displayed seemingly divergent associations depending on the analytical context. When analyzed individually, elevated IL-17A was associated with unfavorable outcomes (relapse after treatment). Yet in the clustering analysis, higher IL-17A concentrations were observed in animals who recovered rapidly, suggesting context-dependent immune dynamics rather than contradictory findings. In animals with rapid recovery, elevated IL-17A concentration may reflect a prompt and effective immune activation that contributes to infection clearance. Conversely, in animals relapsing after treatment, this elevation may indicate a persistent or dysregulated inflammatory response that fails to control infection. These results underline that cytokines such as IL-17A cannot be interpreted in isolation: their prognostic value likely depends on the broader immunological, clinical, and management context. They also support the idea that severe pneumonia is not necessarily associated with a heightened cytokine response. Instead, it may reflect a poor immune responsiveness that is not sufficient to clear infection prior to the onset of tissue damage and clinical signs. Data regarding animals’ medical history, in particular prior BRD episodes, and vaccination status before leaving the farm are currently unavailable in the feedlot production system. This likely contributes to the moderate proportion of cytokine variability explained by the linear models, which accounted for a maximum of 26.3%. Further studies should aim to collect this information to assess its potential impact on cytokine concentrations linked to BRD episodes during the first month of fattening.

Some limitations—including the risk of missing transient cytokine peaks due to weekly sampling and the confounding influence of preconditioning practices such as vaccination and metaphylactic antibiotic use—have already been addressed earlier in the discussion. Nevertheless, it is worth reiterating that these factors likely contributed to the heterogeneity of cytokine profiles observed in this study and should be carefully considered in future investigations aiming to identify reliable immune biomarkers in the context of BRD.

## 5 Conclusion

This study is the first to investigate such an extensive panel of cytokines and chemokines on natural cases of BRD in young bulls during the first month on feedlots. Although individual cytokine and chemokine levels did not allow for clear differentiation between clinically sick and healthy animals, our findings provide novel insights into the complexity and variability of the immune response during spontaneous BRD episodes. Importantly, certain cytokines—namely IL-17A and IFN-*γ—*were associated with disease progression and treatment outcomes, suggesting their potential as prognostic biomarkers, which could be explored in future studies as part of early risk stratification tools for BRD outcome. Beyond the diagnostic and prognostic aspects, this study highlights the importance of preconditioning practices, including vaccination, in shaping the immune response. The influence of these factors suggests that immune status at feedlot entry—and possibly earlier in the animal’s life—plays a key role in modulating the immune response to BRD pathogens. Further studies should aim to integrate detailed data on pre-fattening health and vaccination history. This would help to refine our understanding of the immunological determinants of BRD susceptibility, evolution, and recovery.

## Data Availability

The datasets presented in this study can be found in online repositories. The names of the repository/repositories and accession number(s) can be found at: https://entrepot.recherche.data.gouv.fr/dataset.xhtml?persistentId=doi:10.57745/T2UHEY; https://doi.org/10.57745/T2UHEY.
